# Life-Course Patterns of Work History and Frailty Trajectories Among Community-Dwelling Older Adults in China

**DOI:** 10.1093/geroni/igaf122.191

**Published:** 2025-12-31

**Authors:** Peipei Fu, Yi Wang, Xingzhi Wang, Jiao Yu, Xi Chen

**Affiliations:** Shandong University, Jinan, Shandong, China (People’s Republic); Yale University, New Haven, Connecticut, United States; Duke University, Durham, North Carolina, United States; Yale University, New Haven, Connecticut, United States; Yale University, New Haven, Connecticut, United States

## Abstract

Diverse work experiences in early and midlife stages are associated with physical frailty in later life. However, whether life-course patterns of work history are associated with later life frailty trajectories in China remains unknown. This study utilized data from the China Health and Retirement Longitudinal Study and included 5,133 participants aged 60 years or older. We used sequence analysis to identify the work-history patterns of women and men respectively between age 18 and 60, and conducted group-based trajectory modelling (GBTM) to classify subsequent frailty trajectories. Multinomial logistic regression analyses were then performed to determine the association between work-history patterns and later-life frailty trajectories. Our analysis identified four work-history patterns for both genders. The GBTM revealed four frailty groups for women (persistently non-frail, low increasing frail, consistently increasing frail, persistently high frail) and three frailty groups for men (persistently non-frail, low increasing frail, persistently high frail). Notably, the non-agriculture to retirement work history was significantly associated with frailty trajectories for both genders. The association was particularly strong for participants in the persistently high frail group, with women (Relative Ratio (RR) = 0.42 [95% CI, 0.18, 0.96]) and men (RR = 0.54 [95% CI, 0.30, 0.97]) showing a lower relative risk of frailty compared to participants with lifelong agricultural work histories. Among women, transitioning from agricultural to non-agricultural employed work history was associated with lower relative risks of being frail. Our study highlights the importance of the duration, sequence and timing of transitions in work history for frailty trajectories among older Chinese.

